# A systematic review of the evidence relating to disclosure of psychological distress by mental health professionals within the workplace

**DOI:** 10.1002/jclp.23339

**Published:** 2022-03-05

**Authors:** Aliya Zamir, Anna Tickle, Rachel Sabin‐Farrell

**Affiliations:** ^1^ Division of Psychiatry and Applied Psychology, School of Medicine University of Nottingham, Jubilee Campus Nottingham UK; ^2^ Derbyshire Healthcare NHS Foundation Trust Nottinghamshire UK; ^3^ Nottinghamshire Healthcare NHS Foundation Trust Nottingham England

**Keywords:** disclosure, mental health professionals, psychological distress, stigma, workplace

## Abstract

**Objective:**

To systematically review evidence regarding prevalence and choices of disclosure of psychological distress, by mental health professionals within the workplace.

**Methods:**

Six databases were searched in June 2020. Studies were included if they were published in English language and included empirical quantitative, qualitative or mixed‐methods data. Studies were excluded if they focused on general healthcare professionals or the general population, or on stress or physical health problems. Study quality was assessed using the Mixed Methods Quality Appraisal tool.

**Results:**

Nine studies, with a total of 1891 participants, were included. Study quality varied, with studies generally reporting descriptive surveys using hypothetical disclosure scenarios. Distress was often conceptualized in psychiatric terms. These limitations mean conclusions should be treated with caution. Individuals were less likely to disclose in work and had negative experiences of doing so compared to social circles. Fear of stigma inhibited disclosure. There were differing levels of disclosure relating to recipient, trust, quality of supervision, how distress was conceptualized, and type of problem. Disclosure was experienced by some as valuable.

**Conclusion:**

There is a need for further research, which addresses the nuanced complexities surrounding disclosure choices for mental health professionals.

## 1 INTRODUCTION

Disclosure has been defined as an interaction between two individuals in which one shares personal information about themselves to the other person (Greene et al., 2006). Models of disclosure suggest that decision‐making involves coping with dialectical dilemmas and weighing up the risks and benefits of disclosure (e.g., Disclosure Decision‐Making Model [DD‐MM], Greene et al., 2006; Petronio, 2003). Disclosure of stigmatized identities (e.g., mental health problems [MHPs]) or “mental” distress at work, is a complex decision. It may be beneficial and enable individuals to seek care, gain adjustments and support (Brohan et al., 2014; Corrigan et al., 2016), however, it may also risk stigma and discrimination (Peterson et al., 2011). While employers are prohibited from discriminating against individuals who are experiencing MHPs under the UK Equality Act 2010 and the Americans with Disabilities Act 1990, not all individuals who are distressed may describe their distress in terms of “mental impairment” (Irvine, 2011), as defined by this legislation, thus leaving them unprotected from disability discrimination. Employers are also only able to make adjustment if the “impairment” is known to them. As such, individuals experiencing distress may need to carefully consider their disclosure choices.

Literature on disclosure often uses psychiatric language in defining and understanding human distress which is reflective of the dominance of the “medical model” (Johnstone, 2014). However, this reduces human suffering to categories and symptoms, lacking acknowledgment of the wider social, cultural, political, and psychological influences on human distress (Johnstone & Boyle, 2018). The way in which mental health (MH) is conceptualized, indeed, may also impact upon disclosure prevalence and choices (Cvetovac & Adame, 2017; Irvine, 2011), therefore the inclusion of studies that use both medicalized (e.g., MHPs/diagnoses) and nonmedicalized (emotional/psychological distress) terms is important within this review. The review is also focused on psychological distress generally and not only when considered an “impairment” in terms of disability legislation.

A disclosure model which specifically aims to explain decision‐making processes among people with concealable stigmatized identities is the Disclosure Processes Model (DPM) (Chaudoir & Fisher, 2010). The DPM highlights five main components of the disclosure process including antecedent goals, the disclosure event, mediating processes, outcomes, and a feedback loop. The model posits that approach versus avoidance motivations may underlie disclosure behaviors. While the DPM provides a more holistic account of disclosure processes than the DD‐MM by including mediating mechanisms, it draws on wide‐ranging literature on stigmatized identities, such as sexuality, which may limit its applicability to the processes and functions of disclosures relating to MH.

Much of the research on MH stigma and disclosure in the workplace has focussed on the general population (Brohan et al., 2014, 2012; Toth & Dewa, 2014), however, MH professionals are just as likely, if not more, to have lived experiences of distress and/or MHPs (Brooks et al., 2002; Elliott & Guy, 1993). Indeed, MH professionals with experiences of adversity and distress may be more drawn to pursuing a career in MH (Aina, 2015). Working in MH also risks greater exposure to trauma narratives which may exacerbate MHPs and distress (Engle et al., 2017). A previous systematic review on workplace disclosure within the general population suggests that reasons for nondisclosure at work were fears or experiences of discrimination and stigma, and reasons for disclosure were related to gaining support and adjustments, and being a “role model” for others (Brohan et al., 2012). Among MH professionals, levels of disclosure at work has been found to be related to recipient type, type of MHP, and whether difficulties are current or historic (Grice et al., 2018). However, this study focussed on hypothetical disclosures and MHPs, which may not necessarily reflect actual disclosure choices or experiences. Fear of stigma and negative impact on career are also commonly reported factors that prevent MH professionals from disclosing their difficulties at work (Somers et al., 2014; Tay et al., 2018).

One of the reasons disclosure decisions are complex for MH professionals is because they are bound by ethical guidelines regarding their ability to practice safely. Taking psychologists as an example, the American Psychological Association Code of Ethics (2017) includes Principle A: Beneficence and Nonmaleficence, which states “Psychologists strive to be aware of the possible effect of their own physical and mental health on their ability to help those with whom they work” (American Psychological Association, 2017, p. 3). Furthermore, the same code's section on “competence” includes a requirement that when psychologists “become aware of personal problems that may interfere with their performing work‐related duties adequately, they take appropriate measures, including determining whether they should limit, suspend, or terminate their work‐related duties” (p. 5). Similarly, psychologists in the United Kingdom are required by the Health and Care Professions Council to “make changes to how you practise, or stop practising, if your physical or mental health may affect your performance or judgment or put others at risk for any other reason” (Health and Care Professions Council, 2016, p. 8). While such ethical principles are imperative, it is possible that they may influence disclosure due to concerns that others may deem mental distress as automatically implying a need to significantly change or cease practice.

Stigma is a complex process and occurs when a specific attribute is considered as deeply discrediting within society (Goffman, 1963). It enables a range of social inequalities and discrimination to occur (Parker & Aggleton, 2003), and is evident within structural frameworks of society (Feldman & Crandall, 2007). People may internalize stigma and this can diminish one's sense of self and identity, causing psychological harm (Corrigan et al., 2008). While national efforts to reduce the stigma of MHPs have progressed (e.g., Time to Change, 2008) and have shown positive effects (Evans‐Lacko et al., 2014), there is evidence to suggest that people with MHPs are continually stigmatized within societies (Cunningham et al., 2016; Roskar et al., 2017). Having the opportunity to disclose if desired is important, as concealing difficulties may impact identity integration (Richards et al., 2016), and increase low mood, isolation, demoralization, feelings of shame, and of being different (Link et al., 2001). de Hooge et al. (2010) suggest that shame may activate both approach and avoid behaviors, thereby impacting disclosure choices. Approach behaviors act to restore the threatened self and avoid behaviors serve to protect the self from further damage. Concealing MHPs can also cause strain and emotional stress, which may exacerbate MHPs (Keith, 2013), whereas enabling disclosure opportunities may enable access to support, adjustments, and improve well‐being (Frattaroli, 2006). Given the complex structures surroundings disclosure and the lack of research among MH providers, research within this population is important.

There is a growing body of work and interventions that aim to promote open discussions about MH disclosure and choices in the workplace (e.g., Honest Open Proud [HOP] program) (Corrigan et al., 2013). While this program is a step towards encouraging discussions around disclosure, it focuses upon the general population and self‐stigma specifically. The HOP program has been adapted for MH professionals by University College London (Scior, 2017). As well as disclosure decision‐making, this project aims to encourage open conversations within the MH professions about stigma and lived experience of MHPs, aiming to in turn tackle the “us and them” dichotomy, where professionals may be seen as relatively powerful and clients relatively powerless (Richards et al., 2016).

It is possible that the coronavirus (COVID‐19) pandemic may also lead to changes in disclosure of distress by MH professionals, as the consequent additional pressure on their MH is recognized (e.g., Joshi & Sharma, 2020; Kar & Singh, 2020). Studies specifically about disclosure by MH professionals in the workplace, in the context of COVID‐19 are not yet available. However, Billings et al. (2021) found that their sample of 28 MH professionals supporting frontline health and social care workers during COVID‐19, reported working in isolation, with blurred boundaries and anxiety. However, nearly all subjugated their own MH needs, in part because of guilt about prioritizing their own needs. Many laughed when asked what support they had put in place for their own MH needs and all, but one said they had not considered seeking help for their own distress. Although help‐seeking is not completely interchangeable with disclosure, these findings hint at an absence of disclosure by MH professionals, even in a context of widely recognized and increased MH needs.

Notwithstanding the ongoing efforts to reduce stigma and promote open discussions about disclosure, a detailed systematic review which provides a critical appraisal of the literature has not yet been conducted. Given that much of the previous literature has focussed on the general population, MH disclosure prevalence and choices within the MH professions remain unclear. Much of the previous literature also focuses on psychiatric conditions and it is of importance to include studies that focus on both psychological distress and psychiatric diagnoses within the current review. The current review aims to synthesize and critically appraise the literature on the prevalence and choices related to MH disclosure among MH professionals within the workplace.

### 1.1 Aims

The aims of the current review were to systematically identify, appraise the quality of, and synthesize the evidence regarding:

- The prevalence of disclosure of psychological distress and/or MHPs among MH professionals within the workplace.
- How MH professionals respond to disclosure of psychological distress and/or MHPs within the workplace.
- The choices related to disclosure or nondisclosure of MHPs and/or distress in the workplace.

## 2 METHODS

### 2.1 Design

This review largely followed the Preferred Reporting Items for Systematic Reviews and Meta‐Analyses (PRISMA) guidelines (Page, McKenzie, et al., 2021; Page, Moher, et al., 2021). While they are relevant for mixed‐methods reviews, the PRISMA guidelines state that guidelines for the synthesis of qualitative data should also be consulted. The PRISMA guidelines require assessment of “risk of bias,” rather than broader “quality assessment” or “critical appraisal.” However, despite debates and a range of approaches to conducting it (Garside, 2014), quality appraisal is a standard feature of qualitative systematic reviews and was therefore undertaken rather than a narrower assessment of risk of bias.

A critical realist epistemological position was adopted, which acknowledges the existence of an observable reality but views reality as constructed through our individual standpoints, privileges, meanings, social contexts, and perceptions (Creswell & Plano Clark, 2011). The authors acknowledge that their own positions, experiences, and biases may shape interpretations of the findings. The first author (A. Z.) is a trainee clinical psychologist and has worked in both private and public MH sectors. The author noticed differing responses to workplace disclosures of distress by colleagues and supervisors. The second and third authors are clinical psychologists who work within clinical settings and clinical psychology training and have supervisory and management roles, in which they may receive disclosures of distress. The first author kept a reflective diary throughout the review process and discussions between the three authors were used to check and manage biases in interpretations of the results.

### 2.2 Search strategy

Articles were searched for in PsycInfo, MEDLINE, EMBASE, Web of Science, Grey Literature, and ProQuest Dissertations and Theses in June 2020. Including Grey literature was important to allow for the inclusion of unpublished studies and developments within the field, which may otherwise have been missed. These databases were selected because they were most relevant to the topic of interest in the current review. It was beyond the scope of this review to include all MH diagnoses search terms. Anxiety, depression, and schizophrenia were most commonly used within previous MH disclosure literature (Brohan et al., 2012; Grice et al., 2018), therefore these terms were included over other diagnoses. In addition, terms such as “emotional distress” and “psychological distress” were included within the search strategy, so that relevant studies were not excluded. The term “impairment” was not included because the term offers protection against discrimination under disability legislation in both the United Kingdom (Equality Act, 2010) and the United States of America (Americans with Disabilities Act, 1990). Although such legislative protection cannot eliminate discrimination, it may influence the likelihood of disclosure for individuals who describe their distress in terms of “impairment” and “disability,” compared to those who do not, as well as the likely responses of employers. Given the interest in broader conceptualizations of psychological distress, “impairment” was not included.

The search terms used in PsycInfo are outlined in Table 1. These terms were tailored for each database to ensure suitability in relation to database‐specific thesaurus terms. A published date limit was not applied to ensure that relevant studies were not excluded. The terms were combined using the Boolean terms “OR” and “AND” to search for studies including all three disclosure, MH professionals in workplace and MHPs related terms.

**Table 1:** Literature review search terms

Disclosure	Mental health professionals within workplace context	Distress/MHPs
disclos* conceal* nondisclos* secrecy self‐disclosure	“mental health” adj2 clinician* OR worker* OR therapist* OR personnel* OR practitioner* OR nurse* counselor* counsellor* psycholog* psychiatr* occupation* job employ* work workplace	psychological distress emotional adj2 distress* OR difficult* OR problem* OR suffering* OR disorder* psych* adj2 illness* OR disorder OR diagnos* OR problem* OR disabilit* “mental health” adj2 problem* OR difficult* OR disabilit* OR disorder* OR issue* mental disorder* mental illness* anxiety depress* schizophren*

Abbreviation: MHP, mental health problem.

Studies were selected based on the following criteria:

Studies were included if they:

- Related to the prevalence of disclosure of psychological distress or MHPs among MH professionals within the workplace.

- Related to how MH professionals respond to disclosure of psychological distress or MHPs within the workplace.
- Focussed on the choices related to disclosure or nondisclosure of MHPs or distress in the workplace.
- Related to workplace contexts (paid, voluntary, part‐time, full‐time, private, and public sector).
- Included empirical data (quantitative or qualitative).
- Included data about MH professionals that could be independently extracted from datasets including other groups.
- Were published in English.

Studies were excluded if they:

- Did not focus on disclosure within a workplace context.
- Focussed on general healthcare professionals or the general population.
- Focussed on stress or physical health problems.

Studies relating to both hypothetical and actual disclosure/nondisclosures were included within the search. While it is not clear how much actual and hypothetical disclosures may vary (Bell et al., 2011), excluding studies relating to hypothetical disclosures may have resulted in losing relevant data on disclosure choices among MH professionals. One study, which specifically used the terminology “help‐seeking” was included because it appeared to use this interchangeably with “disclosure” (4). Furthermore, to seek help one is required to disclose their difficulties (Pederson & Vogel, 2007) therefore this study was judged as relevant and included within the review.

### 2.3 Data abstraction and synthesis

Information relating to study characteristics, including lived experiences of distress/MHPs (if reported), prevalence of disclosure, and choices related to disclosure or nondisclosure was abstracted for all studies by first author A. Z. A meta‐analysis was not suitable for the quantitative studies within this review due to the descriptive nature of studies and the heterogeneity of outcome measures and participants (Boland et al., 2017). For qualitative studies, all text related to disclosure prevalence or choices was extracted and analyzed using thematic synthesis (Thomas & Harden, 2008). First data pertaining to prevalence and choices were coded into “free codes” and then into “descriptive” themes. Similarities and differences between codes were identified and “analytic” themes, were developed by A. Z. The analytical themes were discussed and reviewed with the wider research team. For the one mixed‐methods study, both the quantitative and qualitive data were extracted and analyzed according to the description above.

Quotations from the reviewed studies are included in the results section to illustrate the themes. However, it is acknowledged that the present authors cannot determine the criteria the original authors used to determine which quotes they included from their data set.

### 2.4 Quality appraisal

A range of tools and checklists are available to appraise the quality of qualitative research, but given the review included both qualitative and quantitative studies, the quality of studies was appraised using the Mixed Methods Appraisal Tool (MMAT) (Hong et al., 2018). The MMAT has good validity and reliability (Pace et al., 2012; Pluye et al., 2009). The five subsections of the MMAT each provide quality appraisal statements for quantitative studies (randomized control trials, nonrandomized comparative studies, and descriptive studies), qualitative studies and mixed‐methods studies. For the current review, the “descriptive” subsection for the quantitative studies was used, as well as the qualitative and mixed‐methods subsections.

## 3 RESULTS

**Figure 1:**
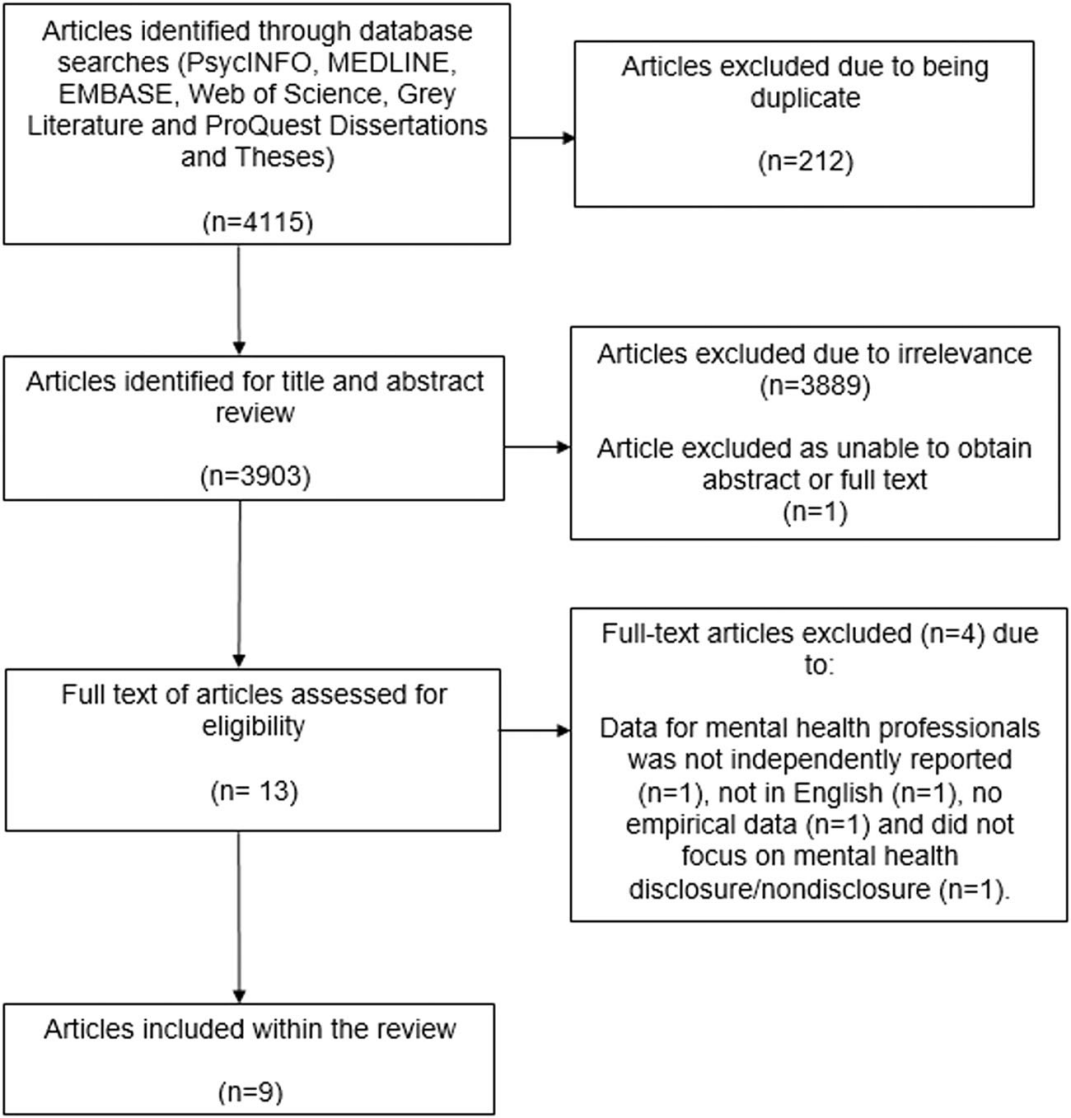
PRISMA flow diagram. PRISMA, Preferred Reporting Items for Systematic Reviews and Meta‐Analyses

The database searches yielded 4115 results. A total of 212 titles were excluded due to being duplicate and 3889 due to irrelevance. The library and author were contacted for the one article where the full text could not be obtained. The paper copy of the study was locked at their university library and inaccessible due to COVID‐19. There was no electronic copy available. A total of nine studies were included in the review after reviewing against the eligibility criteria. Figure 1, based on the PRISMA (Page, Moher, et al., 2021) below summarizes the selection process.

### 3.1 General characteristics

Table 2 outlines the characteristics of all studies. Six were quantitative (4, 5, 6, 7, 8, 9), two were qualitative (2, 3) and one used a mixed‐methods approach (1). The total number of participants across the quantitative studies was 1891. The total sample within the qualitative studies was 22, and the mixed‐methods study had a sample of 77. This resulted in an overall sample of 1990 across all the studies. Two of the nine studies were conducted in the United Kingdom (5, 8), five in the United States of America (1, 2, 3, 7, 9), one in Canada (6), and one in Australia (4). MH professionals within samples included MH nurses, psychiatrists, clinical psychologists, psychotherapists, and psychology faculty staff. The current review focused on MH professionals, rather than students, who may have different issues around disclosure compared to practicing professionals. No exclusion criteria were included as it was assumed that the search terms would identify MH professionals rather than student‐specific studies. However, it is important to note that one study (4) included students (approximately 30% [*N* = 31] of the total sample [*N* = 98]). It was not possible to separately extract data relating to students versus qualified professionals and, on balance, it seemed more important to include the study results in this review than to exclude the views of the majority proportion of practicing MH professionals. It is also noteworthy that another study (5) focused on UK trainee clinical psychologists. Although in a training position, this group hold a dual identity of student and employed, salaried MH professional and thus was included.

**Table 2:** Summary of articles included in the review

Author(s) (year) location	Aims	Sample characteristics Recruitment/sampling	Methodology	Disclosure context	Summary of key findings
1. Boyd et al. (2016) USA	To document lived experience, investigate commonality of disclosure to patients and colleagues and what advice would be given to colleagues with MHPs	MH professionals (psychology [50%], nursing [12%], social work [29%], and other [9%]). Sample size: (*N* = 77) Age range: N.R Gender: N.R Ethnicity: N.R Recruited via email to Veteran Health Administrations groups Purposive sampling method	Mixed methods: Questionnaires Analysis: Descriptive statistics, exploratory analysis, and manual descriptive coding	Actual disclosure experiences of MHPs	Lived experience: Majority reported PTSD, anxiety disorders, depression, bipolar disorder, and psychosis, however exact prevalence N.R Prevalence of disclosure: 31% did not disclose to colleagues, 16% had disclosed to colleagues. People with bipolar disorder had disclosed to a larger number of colleagues than other diagnoses Choices related to disclosure or nondisclosure: 11% of sample advised against disclosing MHPs based on their experiences and 36% reported to be cautious about disclosing. One theme in relation to this was punishment, discrimination, and cruelty (18%) and that stigma still existed. Participants also made comments such as “we are evidence of recovery” and shared hope and strengths in relation to their experiences of MHPs and disclosure
2. Cain (2000) USA	To explore the professional experiences of psychotherapists who have histories of psychiatric hospitalization	Psychotherapists Sample size: (*N* = 10) Age range: 32–57 years Gender: Female (*N* = 7), male (*N* = 3) 32–57 years Ethnicity: All White background Recruited using nonprobability purposive and snowball sampling methods	Qualitative: Semi‐structured interviews and demographic questionnaire Analysis: Thematic analysis	Actual experiences of disclosure/nondisclosure of a psychiatric diagnosis/hospitalization and the impact within the workplace	Lived experience: Primary diagnoses across sample varied, with some having accumulated several diagnoses along the way, however all had experienced MHP(s) at some point in their lives Prevalence of disclosure: Most participants disclosed to colleagues later in their careers. Participants were likely to disclose selectively, and some had never disclosed to anyone at work Choices related to disclosure or nondisclosure: Quality of supervision was related to whether someone had disclosed a MHP and/or hospitalization. Those who disclosed judged their supervisors to be supportive and provide safety. Those who did not disclose, stigma was the main barrier to disclosure and individuals reported a lack of beneficial or quality supervision. All participants reported that the stigma of mental illness was perpetuated within the MH system and for some this hindered their advancement in the profession
3. Cvetovac and Adame (2017) USA	To explore the various meanings of personal distress and how they relate to one's relationships, family, and career	Psychotherapists Sample size: (*N* = 11) Age range: N.R Gender: Female (*N* = 9), male (*N* = 2) Ethnicity: N.R	Qualitative: Published first person accounts Analysis: Narrative	Actual disclosure experiences of emotional distress and psychiatric treatment	Lived experience: All had experienced MHPs at some point in their lives Prevalence of disclosure: Varying levels of disclosure across sample. Exact levels N.R Choices related to disclosure or nondisclosure: All reported fear of stigma and professional repercussions related to disclosure. Themes related to concealment; loss of clinical privileges; being judged as incompetent by supervisors and colleagues. Conflict between desire to open up and being torn about disclosure was common. Some reported positive experiences of disclosing to a colleague or supervisor e.g., it aided capacity to self‐reflect and prevented individuals from becoming overwhelmed by their distress
4. Edwards and Crisp (2017) Australia	To investigate perceived barriers to disclosure and help‐seeking among MH professionals	MH professionals (student, *N* = 31, qualified, *N* = 67). Psychologists (69.2%), other (30.8%) Sample size: (*N* = 98) Age range: N.R Gender: Females (*N* = 82), males (*N* = 16) Ethnicity: N.R Recruited via snowball and purposive sampling methods	Quantitative: Questionnaires Analysis: Descriptive statistics	Anticipated disclosure/barriers to help‐seeking	Lived experience: 40.8% reported experiencing MHPs at some point in their lives Prevalence of disclosure: 64.3% reported that mandatory reporting requirements would prevent them from disclosing to their workplace if they were unwell; 57.1% reported that mandatory reporting requirement would also act as a barrier to seeking help if they were distressed Choices related to disclosure or nondisclosure: Participants reported that they would prefer to get help from friends/family. Concerns related to what people would say at work, embarrassment, and shame. 18.6% reported a barrier of not wanting a MHP on their medical records
5. Grice et al. (2018) UK	To investigate the incidence of MHPs amongst trainees and to understand some of the mechanisms that may underlie their decisions about disclosure	Trainee clinical psychologists Sample size: (*N* = 348) Age range: N.R Gender: Female (*N* = 299), male (*N* = 49) Ethnicity: N.R Recruited via email to 19 UK DClinPsy training course directors Purposive sampling method	Quantitative: Questionnaires Analysis: Exploratory factor analysis, multilevel linear model analysis	Anticipated disclosure of hypothetical MHPs Anticipated disclosure of actual lived experiences	Lived experience: 67% reported experiencing at least one MHP. Anxiety (43%) and depression (39%) were most reported Prevalence of disclosure: Disclosure likelihood varied depending on disclosure recipient. Participants were least likely to disclose a hypothetical MHP to a placement supervisor or course staff member. CS*: Sup −1.53 (MD), −1.68 (Schi), −1.83 (SF), course staff −1.20 (MD), −1.32 (Schi), −1.84 (SF). Participants were more likely to disclose a current MHP, than a past one CS*: 0.23 (MD), 0.35 (Schi), 0.17 (SF), despite anticipating greater stigma with the former. For individuals with lived experiences of anxiety and depression, they were least likely to disclose these to placement supervisors and course staff Choices related to disclosure or nondisclosure: Participants with high levels of maladaptive perfectionism were less likely to disclose a MHP. CS*: −0.03 (MD), −0.03 (Schi), −0.43 (SF). People who indicated high levels of anticipated stigma with a past MHP were less likely to disclose a MHP. CS*: −0.02 (MD), −0.03 (Schi), −0.02 (SF)
6. Hassan et al. (2013) Canada	To assess the attitudes of psychiatrists towards preference for disclosure and treatment, should they develop a mental illness in addition to their own experience of mental illness	Psychiatrists Sample size: (*N* = 487) Age range: N.R Gender: N.R Ethnicity: N.R Recruited via mailing list of CoP and surgeons of Ontario Purposive sampling method	Quantitative: Questionnaires Analysis: Descriptive statistics	Anticipated disclosure of MHPs	Lived experience: 31% reported experiencing a past or current MHP Prevalence of disclosure: 11.1% would disclose to a colleague and 41.9% would disclose to family Choices related to disclosure or nondisclosure: Most important factors related to nondisclosure was commonly reported as “career implications” (34.5%), followed by stigma (23.4%) and professional standing (16.4%)
7. Schroeder et al. (2015) USA	To assess psychologists' responses to a hypothetical situation in which they learn that a MH colleague is seeking personal therapy	Psychologists Sample size: (*N* = 96) Age range: N.R Gender: Female (*N* = 35), male (*N* = 61) Ethnicity: White (92.7%) Recruited via email using online listing of practising psychologists Purposive sampling method	Quantitative: Vignette Questionnaire Analysis: Descriptive and inferential statistics	Psychologists' reactions to fictional vignette with four conditions where colleague discloses; psychotherapy (no disorder specified; psychotherapy for bipolar disorder; psychotherapy for major depressive disorder and control (no psychotherapy/disorder stated)	Lived experience: N/A Prevalence of disclosure: N/A Choices related to disclosure or nondisclosure: Psychologists would continue to refer clients to a colleague who discloses being in personal therapy for MH disorders (depression and bipolar disorder), about as often to a colleague who mentions no disorder/psychotherapy at all. Differences between means did not differ significantly. Referral rate change: (*F* [3, 91] = 2.40, *p* = .073), referral rate delay: (*F* [3, 89] = 0.57, *p* = .639)
8. Tay et al. (2018) UK	To assess the extent to which clinical psychologists report experience of self‐defined MHPs, their views on disclosure and help‐seeking, and to what extent stigma may affectdisclosure and seeking help in relation to MHPs they experience themselves	Qualified clinical psychologists Sample size: (*N* = 678) Age range: Majority (84.2%) 30–50 years Gender: Female (*N* = 557), male (*N* = 121) Ethnicity: White background (91.6%) Recruited via BPS, DCP mailing list Purposive sampling method	Quantitative: Questionnaires Analysis: Descriptive and inferential statistics	Views about disclosure and actual disclosures of MHPs	Lived experience: 62.7% reported experiencing one or more MHP Prevalence of disclosure: Participants most likely to disclose to family (68.2%) than within the workplace (44.5%) (◻^2^ (1) = 26.22).* Most negative experiences of disclosing were to employers. 10.8% had not disclosed to anyone Choices related to disclosure or nondisclosure: Fear of judgment (71.7%), negative impact on career (67.4%) and shame (47.8%) were reported to be the main factors which prevented participants from disclosing MHPs. Those who had not disclosed to anyone showed higher levels of self‐stigma (*M* = 21.860, SD = 6.462) than those who had disclosed at work (*M* = 17.414, SD = 5.571), *d* = 0.737).
9. Zold et al. (2020) USA	To explore faculty members' attitudes toward student disclosures of a history of MH concerns and psychotherapy use in application materials	Faculty staff: Assistant Professor (28.3%); Associate Professor (37.0%); Full Professor (32.6%); and other (2.2%), involved in evaluating student applicants for graduate doctoral programs in clinical and/or counseling psychology. Sample size: (*N* = 184) Age range: 30–72 years Gender: Female (*N* = 99), male (*N* = 85) Ethnicity: White (91.7%), African American (3.9%), Hispanic/Latin(x) (1.1%), Asian (1.7%), mixed ethnic background (1.7%) Recruited via email to faculty programs Purposive sampling method	Quantitative: Vignette of fictional student applicant questionnaires/rating scales Analysis: Descriptive and inferential statistics	MH professionals receiving disclosure Disclosure of depression and/or psychotherapy by a fictional applicant	Lived experience: Fictional applicants disclosing either depression and/or psychotherapy Prevalence of disclosure: 69.8% of staff recommended against disclosing experiences of depression in applications; 64% recommended against disclosing experiences of psychotherapy. Staff from counseling programs and scholar‐practitioner programs were more likely to report that applicants should disclose a history of depression in their application materials. There were no differences in the degree to which faculty recommended disclosing depression or psychotherapy use histories (◻^2^ = 1.31, *p* = .25) Choices related to disclosure or nondisclosure: Faculty members were less likely to accept an applicant who disclosed a history of depression, despite those applicants being rated as equally suited and likely to succeed. Acceptability: *R* = 42, *F* (6, 113) 3.78, *p *= .01, Suitability: *R* = 39, *F* (6, 113) 3.11, *p* = .01, Likelihood of success: *R* = 26, *F* (6, 112) 1.38, *p* = .25

*Note*: Only data pertinent to review aims extracted.

Abbreviations: BPS: British Psychological Society; CoP, College of Physicians; CS, correlation strength; DClinPsy, Doctorate of Clinical Psychology; DCP, Division of Clinical Psychology; MD, major depression; MH, mental health; MHPs, mental health problems; N/A, not applicable; N.R, not reported; PTSD, posttraumatic stress disorder; Schi, schizophrenia; SF, specific phobia; Sup, supervisor.

* Significant at *p* < .001.

Two studies were related to recipients of disclosure (7, 9), whereas all others were related to disclosure/nondisclosure by MH professionals. Most of the studies also included data on lived experiences of MHPs within their samples. Of the quantitative studies, three focussed upon hypothetical disclosure (4, 5, 6) and two on disclosure recipients and their responses to hypothetical disclosures (7, 9). The remaining quantitative study (8), the two qualitative studies (2, 3) and the mixed‐methods study (1) all focussed on actual disclosure experiences. All studies used either survey methods of data collection or a fictional vignette. Apart from one study (2) all were published within the last ten years.

### 3.2 Quality appraisal results

Table 3 summaries the quality appraisal of all studies using the MMAT quality criteria. All nine studies reported on clear research aims/questions and the data collected were appropriate to address the research questions and aims. However, none of the studies drew on existing models of disclosure or relevant psychological theories.

**Table 3:** Mixed methods quality appraisal

	Study number						Comments
*Quality criteria (quantitative studies)*	*4*	*5*	*6*	*7*	*8*	*9*	
1. Is the sampling strategy relevant to address the question?	Yes	Yes	Yes	Yes	Yes	Yes	All sampling strategies were appropriate
2. Is the sample representative of the target population?	CT	CT	CT	Yes	Yes	Yes	Representativeness of samples for half of the studies was unclear
3. Are the measurements appropriate?	Yes	Yes	Yes	Yes	Yes	Yes	All studies used purpose developed measures for variables of interest
4. Is the risk of nonresponse bias low?	No	No	No	No	No	No	There were low response rates across all studies or reasons for nonresponse were not discussed
5. Is the statistical analysis appropriate to answer the research question?	Yes	Yes	Yes	Yes	Yes	Yes	Appropriate descriptive and/or inferential statistics generally used
*Quality criteria (qualitative studies)*	*2*	*3*					
1. Is the qualitative approach appropriate to answer the research question?	Yes	Yes					
2. Are the qualitative data collection methods adequate to address the research question?	Yes	Yes					
3. Are the findings adequately derived from the data?	Yes	Yes					Findings appeared to be logically derived
4. Is the interpretation of results sufficiently substantiated by data?	Yes	Yes					Direct quotes used in both
5. Is there coherence between qualitative data sources, collection, analysis, and interpretation?	Yes	Yes					
*Quality criteria (mixed methods study)*	*1*						
1. Is there an adequate rationale for using a mixed methods design to address the research question?	Yes						
2. Are the different components of the study effectively integrated to answer the research question?	Yes						
3. Are the outputs of the integration of qualitative and quantitative components adequately interpreted?	Yes						
4. Are divergences and inconsistencies between quantitative and qualitative results adequately addressed?	Yes						No divergences or inconsistencies apparent
5. Do the different components of the study adhere to the quality criteria of each tradition of the methods involved?	No						The sample was unrepresentative

Abbreviation: CT, cannot tell.

### 3.3 Quantitative studies (including the quantitative aspect of the mixed‐methods study)

Studies 4, 5, 6, 7, 8, and 9 used nonprobabilistic sampling strategies (purposive sampling) to address the research questions. One study additionally used snowball sampling method (4). A further study used a convenience sample (1). It was not possible to determine the representativeness of the sample for three studies (4, 5, 6) as the demographics and inclusion/exclusion criterion was not clear or present, or because the reasons why people did not respond or the differences between responders and nonresponders were not known. Three studies (7, 8, 9) were judged to have a representative sample as the sample appeared broadly in line with the demographics of that population. The mixed‐methods study (1) was judged to have a nonrepresentative sample as the sample was sought from a pre‐existing group of MH professionals and the author stated it was not representative.

Most studies used purposefully developed disclosure measures which appeared appropriate for the research aims and questions. Studies used single item questions (1, 4, 6, 7, 9) or Likert scales (5, 8). Four studies (4, 5, 8, 9) used validated measures relating to pre‐established variables of interest, for example, concealment, stigma, or barriers to help‐seeking. One study did not state a response rate (1), one study stopped collecting data at the point it met their power calculation (9) and the remainder of studies had response rates of less than 40%.

### 3.4 Qualitative studies (including the qualitative aspect of the mixed‐method study)

All studies used sources of data (participants and recruitment settings) which addressed their research questions and aims and the approaches to data collection were relevant and appropriate. One study did not discuss their findings in relation to the context clearly (2) however the other two studies clearly discussed their findings in relation to the context. One study briefly mentioned their own subjective biases and its implication on the findings and interpretations of results (3). The other two studies did not mention how the author's biases may have influenced the interpretations of findings.

### 3.5 Synthesis of quantitative data

#### 3.5.1 Lived experience

Studies 4, 5, 6, and 8 reported on the prevalence of MHPs or distress within their samples. Prevalence rates of current or past MHPs within the samples were 40.8% (4), 67% (5), 31% (6), and 67% (8). Studies 4 and 6 reported on future hypothetical likelihood of disclosure of distress. Study 5 reported on hypothetical disclosure of hypothetical MHPs (schizophrenia, depression, and specific phobia) and additionally hypothetical disclosure of lived experiences of anxiety and depression by those who had reported these difficulties. Within the study that investigated actual disclosure (8), MHPs commonly reported were anxiety, depression, phobias, and posttraumatic stress disorder. Interestingly, the study that reported lowest levels of MHPs had a sample of all psychiatrists (6), and the one with the highest had a sample of all psychologists (5). Within the two studies that focussed on recipients of disclosure (7, 9), the fictional applicant/colleague was reported to disclose psychotherapy and/or depression or bipolar disorder. The mixed methods study did not outline a specific level of prevalence of MHPs however stated that “most” of their sample had lived experiences of MHPs.

#### 3.5.2 Prevalence of disclosure

One study which focussed on hypothetical likelihood of disclosure (5), reported that recipient type was correlated with likelihood of disclosure. A further three studies on hypothetical disclosure reported that MH professionals would be more likely to disclose MHPs within their social circles, than their work circles (4, 6, 7). One study that focused on actual disclosure, reported that 37.9% of participants had disclosed their MHPs to colleagues or peers, and 25.6% to employers (8). Participants reported greater negative experiences of disclosing to employers, than to family/friends (8). Over half of the sample within this study had over 10 years of experience postqualification (54.3%). Another study which discussed hypothetical disclosure, reported that the Australian Health Practitioner Regulation Agency (AHPRA) mandatory reporting requirement, in which the agency need to be made aware if any practitioner is experiencing a MHP that may impact adversely on their practice, would prevent them from disclosing distress to their workplace if they were unwell (64.3%), and this would also act as a barrier to seeking help (57.1%) if they were distressed. It is difficult to make inferences on the general likelihood of disclosure for this study, as disclosure prevalence was not reported independently from the AHPRA mandatory requirement.

#### 3.5.3 Choices related to disclosure or nondisclosure

From the studies that discussed hypothetical disclosures (4, 5, 6), fear of stigma and negative career implications were consistently reported as reasons for likely nondisclosure of MHPs. For actual disclosures participants reported that they had experienced stigma, exclusion, and discrimination and disclosure had a negative impact on their careers (1, 8). For participants with lived experiences, embarrassment, shame and being viewed as “weak” was reported to prevent them from disclosing (1, 8). Participants who had not disclosed to anyone showed higher levels of self‐stigma, than those who had disclosed (8). Study 5 showed similar results where high levels of self‐stigma was associated with low levels of anticipated disclosure at work (5). Participants within this study were all trainee clinical psychologists, undertaking a doctoral psychology training program.

Likelihood of disclosure of a current MHP was greater than that of a past one, despite their being greater anticipated stigma for the former (5). Participants reported higher levels of stigma for schizophrenia, than depression and specific phobia within this study. Anticipated disclosure of schizophrenia and depression to course staff or supervisors, was reported to be higher than specific phobia (5). The authors reported that this finding was consistent to previous evidence around greater willingness to disclose more heavily stigmatized conditions such as schizophrenia, than anxiety for example, which may be easier to conceal (Brohan et al., 2012).

#### 3.5.4 Recipients of disclosure

Study 9 found that faculty members viewed applicants who disclosed depression on their application forms, as less likely to be accepted onto a psychology course, despite those applicants being rated as equally suited and likely to succeed (9). Faculty members advised against disclosure of a MHP and/or psychiatric treatment on application forms for psychology doctoral programs (9). Study 7 found that if a colleague disclosed a MHP and/or psychiatric treatment to them, this disclosure would not change their behaviors in relation to how much and often they would refer clients to that colleague. Both these studies used hypothetical examples and the vignette and measurement used within one study (7) was brief (two questions). Respondents made comments about wanting more information about the colleague or having a discussion with a colleague to make a more informed choice about how they would respond (7).

### 3.6 Synthesis of qualitative findings

The participants within two studies all had lived experiences of distress, psychiatric hospitalization and/or MHPs (2, 3). The third study reported that most participants had experiences of MHPs, but a figure was not reported (1). The following themes relating to disclosure choices were identified:

#### 3.6.1 Differing levels of disclosure

Levels of disclosure differed across studies and was related to the type of recipient, trust in recipient, quality of support/supervision, the ways in which distress was conceptualized and whether the difficulty was historic or current. In Study 2 it was reported that psychotherapists disclosed their distress later in their careers rather than when they were distressed. This contrasts with the finding in the quantitative study where participants reported that they were more likely to disclose if the problem was current rather than historic (5).

Study 3 reported that when distress was described in relation to life trauma rather than illness, individuals were more likely to feel comfortable to talk openly with their manager about their distress. Within all studies participants were selective and cautious in disclosure (1, 2, 3). One person reported: “Be cautious about disclosure to administrators, there was little support in my experience” (1, p. 615). Another person reported: “I tell my three supervisors only a very small part of my story—that I have lost an important relationship in my life and have been through a time of intense grieving” (3, p. 355). Participants who disclosed their difficulties, stated that they received quality supervision and support, which helped them in disclosing. Those who did not disclose reported less beneficial supervision (2). The finding of recipient type being correlated with disclosure was consistent with quantitative findings, however the qualitative studies (2, 3) provide reasons as to why this may have been the case (e.g., trust in/support from recipient).

#### 3.6.2 Perceived versus actual experiences of stigma, discrimination, and negative impact on career

Where reported, participants who chose not to disclose their difficulties at work, reported that fear of stigma and beliefs that stigma of MHPs was perpetuated within the MH system, prevented them from disclosing. One participant reported: “The culture is still to hide it” (1, p. 615). Other participants also reported fears of being judged as incompetent by their colleagues and supervisors as a barrier to disclosure (2, 3). This is consistent with findings from quantitative studies (5, 8). Participants also reported conflict between their desire to open up but feeling compelled to hide their difficulties. In addition, participants shared that they did not only have to manage the impact of their distress but also the distress of hiding part of their identities, which was reported within one study to be exhausting (3). One participant reported:

I am tired of hiding, tired of misspent and knotted energies, tired of the hypocrisy, and tired of acting as though I have something to hide. One is what one is, and the dishonesty of hiding behind a degree, or a title, or any manner and collection of words, is still exactly that: dishonest. Necessary, perhaps, but dishonest (3, p. 356).

Fears of exclusion, discrimination and negative impact on career were also reported in Studies 1 and 3 and this was a barrier to disclosure. One participant reported: “I have concerns about how disclosure might impact my future when I decide to apply for other jobs” (1, p. 615). Similar fears were also reported in quantitative studies (4, 5, 6).

Within all three studies where participants had disclosed their difficulties at work, it was reported that disclosure impacted them negatively in their professional careers. One participant shared:

I have already lost scholarships, fellowships, and clinical opportunities by being honest about my history. I am not naïve about truth‐telling in a clinical context. I have learned well how important it is to keep the realms of wellness and sickness separate (3, p. 355).

The need to keep wellness and sickness separate, may however perpetuate the “us and them” dichotomy within the workplace. In addition, this may prevent integration of identities, (e.g., service user and professional; Richards et al., 2016). Negative experiences of disclosure at work were also found in the quantitative study 8, however the specific reasons for why participants perceived their experiences of disclosure as negative was not reported. It is therefore unclear whether these negative experiences were related to specific negative outcomes or not.

One individual suggested how stigma may be reduced within the profession: “…administrators and university professors in the MH fields should spend at least 4 h a week interacting with patients” (2, p. 27). However there was no evidence discussed in relation to how spending 4 h with patients has an impact on reducing stigma. A further participant suggested that normalization of MH difficulties can be helpful in reducing stigma, without specific reasons or evidence related to this: “Most people experience anxiety or depression at some point in their life. It needs to be normalized, reduce stigma” (1, p. 615).

#### 3.6.3 Disclosure as valuable

Participants in all studies discussed how disclosure was valuable, for example in helping to model hope and recovery to service users and colleagues in similar situations and being an asset to the profession. One participant reported: “We should embrace the additional skills this brings to VHA” (1, p. 615). Some participants were hired specifically due to their lived experiences (2) and shared how they used their experiences within their work. For example, one person reported: “The most positive impact is… that I really know what's going on for [clients] a lot more deeply than someone who hasn't experienced [mental illness] (2, p. 26).

Similarly, it was reported that disclosure may inspire others to disclose and that perhaps disclosure to someone with similar experiences may be important:

The most important part of my recovery was sitting with someone who I knew had gone that route before…this can be one of the most critical differences in recovery, because basically, the message you [usually] get is that you can't do it, and to actually see someone there who's done it [is valuable]. So, I just say that sort of is my plea to the field, that it's really important to reduce the stigma and open up this opportunity… (2, p. 27).

## 4 DISCUSSION

This review aimed to synthesize the evidence on the prevalence and choices related to disclosure of psychological distress and/or MHPs among MH professionals within the workplace. This review has provided a critique of the existing literature and found that studies have been of varying quality. This review also found that the literature has lacked the depth and nuanced understanding of the complex processes involved in disclosure choices and decisions, which draws on relevant psychological theory (e.g., stigma, shame). Shame may activate approach or avoid behaviors (de Hooge et al., 2010) and disclosure may be mediated by approach or avoid motivations (Chaudoir & Fisher, 2010). Few studies discussed these complex processes, even though shame and stigma were found to be related to disclosure choices for MH professionals. There perhaps need to be specific investigations of the relevance and applicability of existing models and theories, to workplace MH disclosures by MH professionals. Most quantitative studies in the current review used survey methodology, which loses the context in which disclosure may or may not occur. The small number of qualitative studies also lacked discussion of findings within context, and how the authors' biases may have impacted upon study findings and interpretations. Therefore, the results in relation to prevalence and choices of disclosure should be treated with caution. In addition, most studies did not seek evidence for actual disclosure experiences and used hypothetical likelihood of disclosure and/or responses to disclosure scenarios. Whilst this evidence is useful, it is unclear whether this would reflect the actual choices and outcomes for MH professionals (Bell et al., 2011). The lack of research on actual disclosures may also reflect researcher bias, and potential assumptions of researchers that MH professionals are reluctant to talk about their distress and disclosure choices. It appears that within studies that included actual experiences, MH professionals were willing to talk about their experiences (1, 2, 8).

The review found that lived experiences of distress or MHPs among MH professionals were common, however this finding may be related to self‐selection bias, as individuals who are more willing to talk about MH disclosure may be more likely to take part in MH disclosure research. MH professionals generally perceived that they would be least likely to disclose their distress within work circles compared to their social circles. In keeping with previous research (e.g., Corrigan et al., 2016, Peterson et al., 2011; Toth & Dewa, 2014), barriers such as anticipated stigma, discrimination and negative impact on career were commonly reported to prevent MH professionals from disclosing MHPs and/or distress at work. For example, self‐stigma was evident across many studies and participants reported that shame, embarrassment and perceiving MHPs as a “weakness,” were barriers to disclosure (4, 5, 8). This finding is consistent with previous MH disclosure literature within the general population, where self‐stigma was commonly reported to prevent individuals from disclosing MHPs at work (Brohan et al., 2012; Corrigan & Matthews, 2003). Participants who had actual experiences of disclosing their distress at work, reported that the stigma of MHPs was apparent within the work culture and disclosure had a negative impact on their career (e.g., loss of clinical privileges) (3). This suggests that structural stigma within workplaces may exist and stigma toward MHPs may be perpetuated within the healthcare system. This may be particularly pertinent to address, given the potential impact of the COVID‐19 pandemic on the MH of clinical staff (Fernandez et al., 2021).

Stigma and labeling are complex processes (Goffman, 1963) and the review found a lack of discussion within studies, on how MH stigma within the workplace might intersect with other axes of disempowerment and marginalization (e.g., race, class, gender; Stangl et al., 2019). For example, for male MH professionals stigma of MHPs and help‐seeking may be more profound due to dominant discourses and social norms around “masculinity” (Möller‐Leimkühler, 2002). Emphasis of these broader constructs within interventions that aim to help MH professionals to cope with stigma and make choices around disclosure, are important. In addition, interventions may seek to shift harmful norms through dialog and engagement with local leaders.

When describing their distress in relation to life trauma rather than illness, participants were more likely to feel comfortable in talking openly with their manager about their distress (3). Therefore, it is important to consider how MH and distress conceptualizations may impact upon disclosure choices. In addition, there was greater stigma reported for more heavily stigmatized conditions such as schizophrenia (5). This finding is consistent with previous literature which suggests that greater stigma is associated with psychiatric diagnoses and type of MHP (Angermeyer & Dietrich, 2006). It was also apparent within the results that psychiatrists were less likely to report or disclose lived experiences of MHPs in the workplace, than psychologists. It may be that the differing training and MH conceptualizations within these professions impacts upon disclosure prevalence and choices.

Only a few studies provided insights into the positive outcomes and experiences of disclosure within the workplace. Whilst many studies reported on stigma, stigma may also foster resilience and fuel the formation of advocacy groups (Stangl et al., 2019). Whilst the theme of disclosure being valuable was apparent in the qualitative literature, researchers generally did not specifically look for positive experiences. There is a risk that stigma may be perpetuated due to researcher bias. Whilst some studies suggested some ways in which stigma may be contended with, such as normalization of disclosure of distress (1), there is little evidence to support how this might help reduce stigma. It may be that further investigation into normalization of disclosure and the different functions it might serve, for different people at different times, is warranted. In addition, there is a need to explore workplace disclosure structures that already exist, and support MH workplaces in developing guidance, structures, and pathways which enables opportunities for workplace disclosure if desired by an individual. MH professions may seek to draw upon support resources that are already available. For example, the Mind charity provide free workplace wellbeing plans and guidance for employers and employees in managing and responding to MHPs at work (Mind, 2020). Furthermore, innovations such as the HOP project (Corrigan et al., 2013; Scior, 2017) also exist to promote open discussions about workplace MH and disclosure. It may be that research has not yet caught up with existing innovations. Ongoing research and evaluations of existing innovations and how these may be adapted and utilized within the MH professions therefore seem important.

### 4.1 Limitations

The search process may have excluded relevant studies due to the search being limited to studies published in English and studies that included empirical data. It is also noteworthy that there was a lack of studies from low‐ and middle‐income countries, which is not likely to be explained by restriction to English language publications alone. In addition, only two studies within the review were conducted within the UK therefore the findings may not be generalizable to the UK MH context. There was also an absence of qualitative studies within the review and thus the thematic synthesis was derived from a small number of studies, which may limit the conclusions drawn. Finally, whilst efforts were made to limit biases, inevitably author biases may have impacted upon inferences drawn. Nevertheless, this review has provided a critical insight into the methodological shortcomings of studies within the field and discussed aspects in relation to how the field may be developed.

### 4.2 Future research

Future research should seek to limit researcher and selection bias, given the lack of studies that focussed on positive experiences of disclosure. It is important to investigate the perspectives of MH professionals who have and have not chosen to disclose MHPs/distress in future research, rather than researching views based on hypothetical disclosures. This may involve quantitative, qualitative, or mixed methodologies with a focus on actual disclosure experiences in the context of complex stigma processes. Such processes are arguably more likely to be captured within lived experience rather than hypothetical scenarios, and thus may help to further explain disclosure experiences among MH professionals.

Future research may seek to investigate the concept of normalization of disclosure and the different functions disclosure may serve for different people at different times. The way in which MH distress is conceptualized and how this might impact disclosure choices, seems of particular importance. Further research is also needed in countries not represented by the papers in this review. Researching the specific context of the COVID‐19 pandemic may provide additional understanding regarding disclosure decisions, given the wide recognition of consequently increased MH needs. Finally, it is important for future research to evaluate existing innovations and interventions that promote choices around disclosure.

## 5 CONCLUSIONS

The current review outlines the limitations and strengths of current research within the field and highlights the need for methodologically sound further research which explores and addresses the nuanced complexities around disclosure decisions and choices for MH professionals. The prevalence of disclosure of MHPs and/or distress among MH professionals within the workplace, is much lower than the levels of distress or MHPs MH professionals report. However, these findings may be related to selection and researcher bias. MH professionals report experiences or expectations of stigma, exclusion, and negative impact on career, which is consistent with previous disclosure research (Brohan et al., 2012). There was some evidence within the review that disclosure of distress was valuable however studies tended to generally focus on negative experiences. MH professions have an opportunity to learn from existing disclosure innovations. Whilst initiatives have started to develop specifically for MH professionals, that promote open conversations about MH distress among MH professions and disclosure (e.g. the HOP project; Scior, 2017), research evidence does not align with these innovations.

### PEER REVIEW

The peer review history for this article is available at https://publons.com/publon/10.1002/jclp.23339
